# Design and fabrication of adjustable red-green-blue LED light arrays for plant research

**DOI:** 10.1186/1471-2229-5-17

**Published:** 2005-08-23

**Authors:** Kevin M Folta, Lawrence L Koss, Ryan McMorrow, Hyeon-Hye Kim, J Dustin Kenitz, Raymond Wheeler, John C Sager

**Affiliations:** 1 Horticultural Sciences Department and the Plant Molecular and Cellular Biology Program, University of Florida, Gainesville, USA; 2 Biological Sciences Office, Space Life Sciences Laboratory, Kennedy Space Center FL, USA; 3 Dynamac Corporation, Space Life Sciences Laboratory, Kennedy Space Center, FL, USA

## Abstract

**Background:**

Although specific light attributes, such as color and fluence rate, influence plant growth and development, researchers generally cannot control the fine spectral conditions of artificial plant-growth environments. Plant growth chambers are typically outfitted with fluorescent and/or incandescent fixtures that provide a general spectrum that is accommodating to the human eye and not necessarily supportive to plant development. Many studies over the last several decades, primarily in *Arabidopsis thaliana*, have clearly shown that variation in light quantity, quality and photoperiod can be manipulated to affect growth and control developmental transitions. Light emitting diodes (LEDs) has been used for decades to test plant responses to narrow-bandwidth light. LEDs are particularly well suited for plant growth chambers, as they have an extraordinary life (about 100,000 hours), require little maintenance, and use negligible energy. These factors render LED-based light strategies particularly appropriate for space-biology as well as terrestrial applications. However, there is a need for a versatile and inexpensive LED array platform where individual wavebands can be specifically tuned to produce a series of light combinations consisting of various quantities and qualities of individual wavelengths. Two plans are presented in this report.

**Results:**

In this technical report we describe the practical construction of tunable red-green-blue LED arrays to support research in plant growth and development. Two light fixture designs and corresponding circuitry are presented. The first is well suited for a laboratory environment for use in a finite area with small plants, such as Arabidopsis. The second is expandable and appropriate for growth chambers. The application of these arrays to early plant developmental studies has been validated with assays of hypocotyl growth inhibition/promotion and phototropic curvature in Arabidopsis seedlings.

**Conclusion:**

The presentation of these proven plans for LED array construction allows the teacher, researcher or electronics aficionado a means to inexpensively build efficient, adjustable lighting modules for plant research. These simple and effective designs permit the construction of useful tools by programs short on electronics expertise. These arrays represent a means to modulate precise quality and quantity in experimental settings to test the effect of specific light combinations in regulating plant growth, development and plant-product yield.

## Background

Light drives the processes of photosynthesis and plant development, and ultimately affects crop yield. The culmination of over a century of plant photobiology research shows that plants possess complicated photosensory networks that monitor and respond to a wide spectrum of ambient light energies. The spectral sensitivity of the plant light-sensors greatly exceeds the range of human vision, as light effects on physiology have been observed from energies arising from the UV-B wavebands [1] into the near infra-red [2]. This broad range of environmental information is processed by integrated signaling networks that tailor growth and development to best fit ambient light conditions.

Light sensing pathways have been dissected through use of narrow-bandwidth light sources. Since individual photoreceptors are generally tuned to sense specific regions of the spectrum, narrow-bandwidth irradiation allows isolation of effects associated with a particular waveband. For instance, the phytochromes mediate responses to red and far-red light, with partial activity extended through the green, blue and near-UV wavebands. Cryptochromes are required for maximal response to blue and UV-A [3], while the phototropins exhibit autophosphorylation when stimulated with light qualities from the blue-green interface (500 nm) to UV-C [4]. Other sensors share sensory overlap with the phototropins, and at least several other receptors remain to be characterized [5].

Narrow-bandwidth, research-quality light is typically generated using a broad spectrum source (such as an incandescent lamp) filtered through an infra-red heat sink and several layers of acetate theatre filters or colored plastic. Fluorescent lamps also are used, as they emit three principle light qualities that can be readily filtered to obtain narrow-bandwidth light. LED light has been used principally in studies of phytochrome reversibility, as switchable red and far-red LED arrays are commercially available. Many reports have demonstrated the utility of red/far-red LED sources in modulating phytochrome responses during de-etiolation [6-8], modulation of root growth [9], root greening [10] and senescence [7]. LED technology has been incorporated into lighting regimes to modulate plant growth and development for decades as acute supplementation of sunlight (such as [11-13]) or as the basis of plant growth in commercial chambers (as in [14]). Additionally, LED-generated light is well-suited for small growth chambers and other applications where a significant fluence rate is required, but little physical space is available for conventional lamps. The practical aspects of LED lighting make them particularly well-suited for space applications where light treatments need to be precise and reliable with little heat radiation and low weight. LEDs may also be especially useful in retrofitting incandescent or fluorescent growth chambers for terrestrial applications.

**Figure 1:**
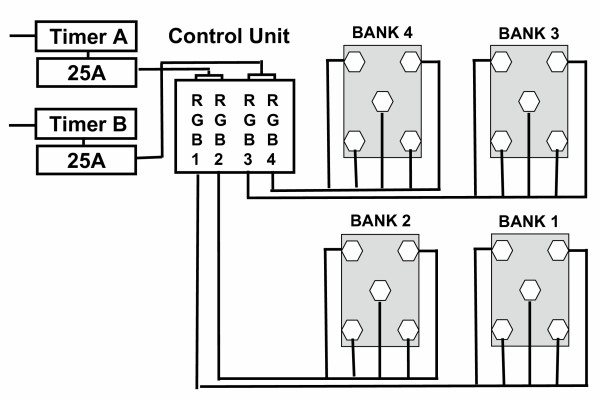
A simplified diagram of Design 1. Here 20 Norlux HEX arrays are configured into four individual banks of five arrays each. R, G and B are individually controllable on each bank, allowing the use of the controller to regulate light conditions over four independent light quality and quantity conditions. The four banks are regulated by two independent power supplies, actuated by two separate timers. In this configuration two separate photoperiods may be tested for any set of experimental parameters.

**Figure 2:**
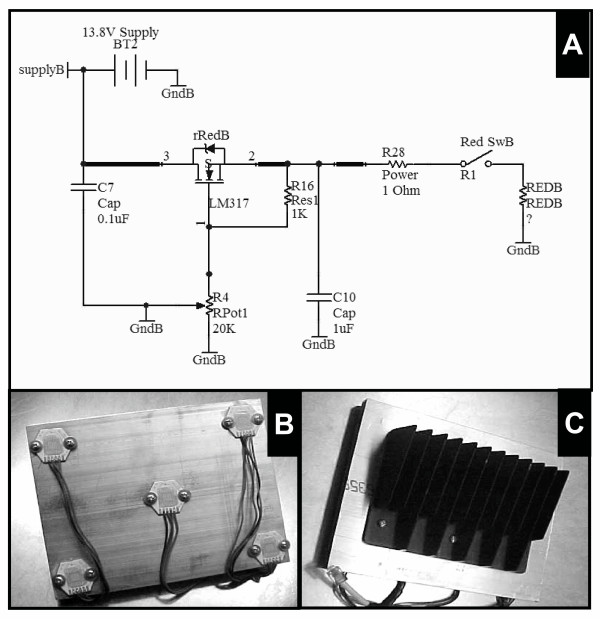
A) The schematic of the Tunable LED controller circuit used in Design 1. The controller regulates the output of four sets of five LEDs. The schematic represents the circuit used to power one color (R, G, or B) in five separate HEX chips, and therefore is repeated twelve times in the controller unit in this arrangement. The 5 HEX LED bank is shown from a bottom (B) and top (C) view.

**Figure 3:**
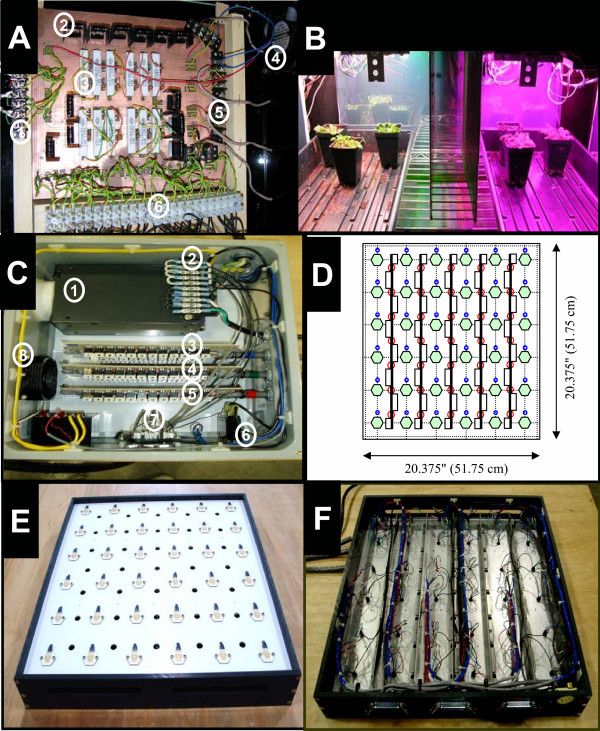
A) The components and organization of the RGB control unit used in Design 1; 1. 12 VDC power supply input bus, 2. voltage regulator array, one regulator to supply one color on five HEX arrays, 3. power resistor array, one for each color on 5 hex arrays, 4. output fan, 5. output bus, 6. potentiometer bus. B) The Design 1 arrays in operation, tuned to an environment without red (left) and without green (right) C) The components of the 36-element control unit; 1. power supply, 2. 12 VDC bus, 3,4,5, red, blue and green circuit boards, 6. on-off switch/breaker, 7. pulse-wave modulator, 8. cooling fan. D) A diagrammatical representation of the 36 HEX LED layout. The position of HEX arrays (green hexagons), wire pass-through holes (red circles), ventilation holes (blue circles), and the vertical heat sinks (black bars) are presented. The complete 36-element array (top; Panel E) and bottom (Panel F).

**Figure 4:**
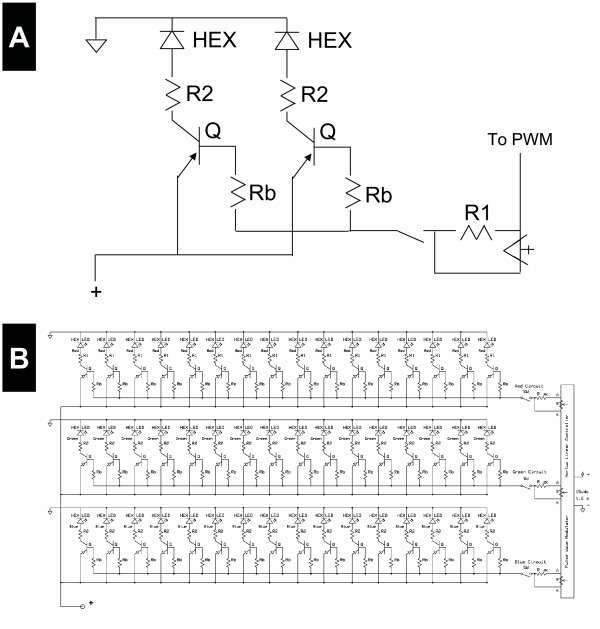
The circuitry powering the large LED banks. A) Two repeat units, driving two independent sets of 30 dies in Norlux HEX arrays. Q = TIP 170 PnP transistor; R1 = 22 ohm, 1 watt, metal film resistor (+/- 5%); R2 = 20 ohm, 1 watt, metal film resistor (+/- 5%); Rb = 4.7 k ohm, ½ watt, metal film resistor (+/- 5%). B) The assembly of the individual units into a controller for red, blue and green circuits in a 16 HEX array.

There is a need for a light source that may facilitate plant growth in a chamber environment, yet still allow for dynamic variation in light quality and quantity for experimental studies. Such devices may reveal interesting interactions between light sensing systems. Recent findings demonstrate that even "benign" wavebands (like green light) have significant influence in plant physiology in concert with red and blue light [15-17]. In this report we detail the design and construction of a compact LED light array for use in plant research. These light sources utilize Norlux hex-LED arrays and are capable of delivering 0 – 150 μmol m^-2 ^s^-1 ^of combined red (R), green (G), and/or blue (B) light. The design allows precise fluence-rate control of individual wavebands, allowing growth of plants under different combinations of light energies. These designs are the same as those used to generate plant-growth data in a number of recent studies of red, blue and green light interaction [17,18]. Two designs are presented in Figures 1, 2, 3, 4. The first is a plan that may be suitable for use in the laboratory or classroom for fewer than one thousand dollars. The second depicts an expandable system that may be appropriate for large growth chambers. Together the two plans presented represent tested and proven designs to introduce efficient lighting to chambers where light quality, quantity, duration and mixture can be readily controlled.

## Results and discussion

The advent of new semiconductor technologies has inspired a marked decrease in the price of LED-based devices. An increased number of consumer-grade products have become available to the researcher, and now these new tools may be integrated into various light-research applications. The goal of this work is to provide an interface between research needs and new technology. With this, the best-available research tools may be implemented by researchers without a significant investment in development. The plans presented herein offer two options for LED light source construction, based on the need of an experimental illumination tool or requirement for large-area irradiation.

LED-based lighting regimes are being adopted by municipalities and medical facilities for their consistent, low-power, low-maintenance output. However, one of the most important practical applications of this technology is in the design for lighting regimes to support plant growth. It is of great interest to not only to foster plant growth, but to *control *plant growth. Basic plant research has demonstrated that specific light wavebands may affect discrete aspects of plant physiology, such as germination [19], stem growth [20], biomass [15,21,22] and the transition to flowering [23]. The supplementation of specific wavebands or skewing of overall spectrum may help modulate the progression of these developmental events. The possibility that combinatorial light regimes may help to optimize growth and control developmental transitions makes the implementation of LED technology particularly attractive to the design of controlled environments targeted to plant production for aesthetic applications, or applications relevant to human nutrition. If spectral quality alone can delay or hasten the floral transition it may have profound effects on modulating the delivery of nursery goods or perhaps affect the availability of consumable produce in a finite, controlled environment. This attribute alone makes LED lighting a compelling platform for specific plant growth routines, such as those proposed for long-term inhabitation of space. Since humans rely specifically on vegetative parts (like stems, leaves and tubers) or reproductive parts (berries and seeds) of plants for nutrition, it is critical to develop systems which impart control of the progression of plant development to affect plant output toward the particular needs of humans.

The implementation of narrow-wavelength LED technology may benefit plant growth schema through supplementation or complete retrofitting of existing chambers. Its compact design may replace existing infrastructure with long-life and consistent output. Here, antiquated lamp systems, replete with toxic, inefficient fluors, may be refitted with efficient light sources that require little to no maintenance with comparable light output. Although previously unattainable without substantial engineering, the geometry of the systems provided in this report brings LED technology to the average plant biology laboratory.

Despite their vast advantages over conventional lighting systems, the LED arrays described in this report offer opportunities for improvement and expansion. Larger installations (e.g. 100 HEX arrays) require close attention to array density, as the fluence rate of RGB HEX LED lights decays significantly toward the edges of the irradiation area. Careful arrangement modified to the application lessens the frequency of "hotspots" or other gradients of light intensities under the light fixture. It is impossible to eliminate all variability under the arrays under all fluence rates and light combinations. The spacing of HEX units in individual systems needs to be carefully tailored for the specific application.

Another potential improvement would be to integrate PAR sensors into the system to provide irradiance feedback and adjust light intensity through a computer-aided regulatory circuit. This would allow the user to enter a specific irradiance value for the desired wavelength and would compensate for changes in LED output that occur over time and with temperature changes in the ambient environment.

This system has been developed using LEDs emitting three principal wavebands. The clear extrapolation is to add additional LED types to generate additional spectrum coverage. LEDs currently manufactured include UV, far-red and infra-red light. From the plans presented within this report it may be possible to develop lighting systems that roughly approximate solar output by compounding the effects of multiple LEDs. Such a system may prove especially valuable in optimizing plant physiology and may have applications to human physiology as well.

**Figure 5:**
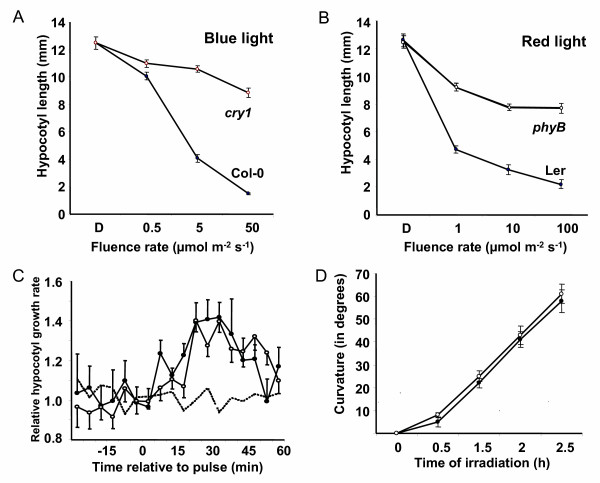
Norlux arrays induce typical light responses during early seedling development. A) Blue light fluence-rate response experiments demonstrate the effect of increased fluence rates of blue light on stem growth after 72 h (filled circles; n = 60). The *cry1 *mutant was tested for comparison (n = 62). B) Fluence-rate/response experiments were performed using red light and demonstrate growth inhibition in wild-type seedlings (filled circles; n = 70) compared to *phyB *mutants (open circles; n = 60). C) The stem-stimulatory effect of a short single pulse of green light is shown (open circles) and compared to previously published results ([17]; filled circles), and dark growth kinetics (dotted line). D) Phototropic curvature in response to blue light was measured in etiolated seedlings (filled circles; n = 21) and compared to results from previous reports ([30]; open circles). These findings demonstrate how the HEX arrays and the designs presented herein have utility in study of integration of light information from multiple photosensory systems.

The arrays described were tested for support of normal plant developmental responses. These are most conspicuous in early seedling development, as initial responses to light are rapid, robust, and have been well characterized [5,24-26]. Three responses to light, namely inhibition of stem elongation (red or blue activated), stem growth promotion (green activated) and phototropism (blue activated), have been extremely well characterized and may be used to test and verify the utility of these LED arrays on early plant developmental responses. Three assays were conducted. First, end-point stem growth was measured in plants grown under three fluence rates of red or blue light. Red and blue light strongly inhibit early stem elongation through the phytochrome and cryptochrome systems, respectively [20,26]. The results of two independent fluence-rate/response experiments using over 60 seedlings are shown in Figure 5A and 5B. Figure 5A shows the height of seedlings grown for 72 h under constant blue light and Figure 5B shows the effect of the same treatment with red light. The *cry1 *and *phyB *mutants are presented as controls. The data show that constant blue or red light inhibit seedling elongation in a manner roughly proportional to fluence rate. Inhibition is detectable even at low fluence rate (<10^0 ^μmol m^-2 ^s^-1^), leading to a strong inhibition of stem growth elongation. The results of these trials mirror the previously published results [27,28], suggesting that the LED arrays described herein act in a manner similar to those used previously.

While phytochromes and cryptochrome effects are salient as stem growth inhibition after days of growth in constant light, other rapid responses involve other light sensors and can be measured on the order of minutes rather than days. Contrary to the effects of red and blue, a short single pulse of green light stimulates rapid elongation of the hypocotyl in the dark-grown seedling [17]. The response persists in all photomorphogenic mutants, it occurs when plants are grown in constant dim red light, and growth promotion is the opposite of what occurs when seedlings are irradiated with red or blue light. This evidence renders it difficult to conveniently ascribe this response to any of the known light sensors, and it is likely being mediated by a separate green-sensitive transduction pathway. Figure 5C, shows the results of 20 independent seedlings treated with green light from the Norlux HEX arrays, compared to previously published data [17]. Seedlings irradiated with a short, single pulse of green light begin to grow rapidly within 15 min, attaining 140% of their dark rate before growth rate declines to dark levels after an hour. The results are highly similar to published findings, again indicating that Norlux HEX arrays are a suitable alternative to other LED or fluorescent light sources.

Phototropism is the rapid curvature of the hypocotyl toward a unilateral light source. In Arabidopsis phototropism is blue light induced and is mediated by the phototropins, autophosphorylating serine-threonine kinases associated with the plasma membrane [29]. The response is exquisitely sensitive to blue light. Here wild-type seedlings were irradiated from the side while being imaged in 5-minute intervals with an infra-red CCD camera (as in [30]). The degree of curvature was monitored over two hours and compared to previous results (Figure 5D). The results indicate that the rapid response of phototropism is very similar between the arrays designed in this study and those produced commercially.

## Conclusion

As the availability of new technologies increases, research programs are challenged with the need to retool their capabilities to exploit their potential. Since many of these new technologies are electronic and/or computer related, a certain degree of technical prowess is required to move ideas from the drawing board to application. The development costs of these technologies may be significant. This report offers two clearly applicable models of LED light source development that may be implemented in the study of plant growth and output, helping to narrow the long-term challenges of illumination to support plant growth. These designs allow the fine control of specific wavebands shown to influence plant growth and development. Such designs represent the first step in defining conditions that will optimize, or perhaps even control, plant growth and development. The light sources used in this report sustain normal plant early developmental responses, suggesting that they are an appropriate substitute for, or complement to, other plant illumination solutions.

## Methods

The NorLux HEX LEDs (Norlux Corp., Carol Stream, IL) were chosen for these applications because of their compact size and power input to output efficiency. The device is a 90-die, densely-clustered set of LED chips, 30 red (628 nm), blue (470 nm) and green (530 nm) in one device. The HEX platform implements chip-on-board technology which allows high fluence rates to be generated from a relatively small ~2 cm-diameter source. The solid-state design is affixed to a ceramic and steel support to facilitate efficient heat transfer to the mounting substrate. These qualities make the Norlux HEX array useful in applications where high fluence rates need to be generated in minimal space, such as a growth chamber or experimental setting. Most importantly, the RGB components may be independently controlled to finely adjust the quality and quantity of irradiance. Two designs of array controller and array construction are presented. The first is the design of the compact 5-HEX arrays, modular and tunable RGB banks at the University of Florida. These were designed for small spaces and use in studies of stem growth and plant development in the model system, *Arabidopsis thaliana*. The second design details the construction of the larger light banks of 36 HEX arrays built for studies at Kennedy Space Center. Here, larger light banks are required to assess the effects of light quantity and quality on the growth of specific crop species likely to be grown in space. Complete parts lists for both array designs are presented in Table 1. All materials are common electronics components and may be obtained through local (eg. Radio Shack) or online vendors [31,32].

### Design 1 – The tunable RGB banks at the University of Florida

Research activities by this research group at the University of Florida center on developmental transitions, namely the progression of seedling growth between dark and light environments as well as the vegetative/floral transition. These processes are all controlled by a well-defined set of photosensors, responsive to the various portions of the spectrum. The ability to independently control specific wavebands makes it possible to assess how independent light sensing systems integrate environmental information to tailor the developmental response in question. Experiments are performed on small seedlings or throughout the life cycle of *Arabidopsis thaliana *plants, so an optimal light bank would be self contained, relatively small, and easily moveable. With these considerations in mind, the tunable RGB banks were developed. Each tunable RGB bank consists of 5 HEX arrays (Figure 1), 150 total dies, where the red, blue and green dies are independently adjustable. 20 HEX arrays allow for 4 independent light banks that may be run simultaneously with equal spectral output to irradiate a large area (~1.0 × 0.25 m). Alternatively, each may be independently controlled to illuminate up to four separate spectral quality/quantity conditions at the one time. Two separate photoperiods may be controlled using two independent power supplies and timers.

### Control unit

The Control Unit (CU) was designed to support twenty HEX units, four sets of controls to regulate RGB independently on five HEX units. By specification, the Norlux HEX array has a maximum input voltage of 11.7 V and 1.5 A of input current (200 mA per HEX). A circuit was developed where a single potentiometer controls the output from each of the red, blue and green LED arrays in 5 separate HEX units. A single circuit that controls one color in five arrays is shown in Figure 2A. This circuit consists of a standard 25 A power supply delivering 13.8 VDC to a common bus that feeds an LM317 voltage regulator. Voltage to the array is modulated by a potentiometer as well as an in-line switch. A 1 K ohm power resistor is placed in the circuit as a current limiter. The voltage regulator and the LEDs require a stable DC input. Input and output capacitors were added to minimize ripple for improved transient response. A more stable voltage waveform assures consistent output. This simple configuration is repeated for each color. There are twelve individual circuits in the control unit, each controlling R, G, or B in each of four light arrays.

In Design 1, the LM317 voltage regulator is used to ensure an output of 1.5 A, which is important to achieve the maximum light output, yet each generates substantial heat. The voltage regulator has a threshold temperature of 125°C, and the twelve regulators used in this design must be equipped with individual heat sinks. The CU was outfitted with two 80 mm 12 V fans, one facing into, and one facing out of the CU. Each of the individual voltage regulators was outfitted with an individual heat sink to assure adequate cooling.

### The RGB bank

Each Tunable RGB Bank consists of five RGB HEX arrays affixed to a 12.5 mm solid aluminum support (Figure 2B and 2C). To allow for modular removal, replacement or service of individual HEX units, the four HEX array connection wires (1 each, RGB anodes and ground) were attached to a standard RJ11 telephone connector. The output of the CU terminates in a 5-input RJ11 coupler where all five HEX arrays could be easily, yet reversibly interfaced.

Thermal issues must be considered in construction of the light source. The LEDs have a maximum temperature rating of 110°C. To maintain full operation without heat damage to components, the NorLux HEX arrays required mounting to a substrate capable of efficient heat transfer and dissipation. In this design, HEX arrays were affixed to solid 18 mm aluminum blocks with heat-sink compound and metal screws (Figure 2B). A black-anodized alloy heat sink was attached to the aluminum block and an 80 mm fan was installed over each unit (Figure 2C). With this configuration the aluminum block temperature did not exceed 45°C. A low operating temperature not only assures consistent LED output, but also is important as radiant heat may perturb plant growth and/or development.

The modular design of the four Tunable LED Banks permits the capacity to control the quantity and quality of actinic light in plant experiments (Figure 3A). In this arrangement up to nine 2.5" (6.35 cm) pots containing rosette plants such as Arabidopsis may be grown under a fluence rate of 75 μmoles m^-2 ^s^-1 ^(+/- 5 μmoles m^-2 ^s^-1^) at 15 cm vertically below from the light fixture (Figure 3B). Alternatively, seedling hypocotyl growth experiments on Petri dishes may be performed at much closer proximity (5 to 7 cm) where a uniform fluence rate of over 100 μmoles m^-2 ^s^-1 ^is readily obtained.

### Design 2 – The large RGB light banks at Kennedy Space Center

Unlike the small 5-array banks described previously, large 36 HEX LED light fixtures were developed to accommodate the uniform irradiation of large flats of plants used in the study of crop production for advanced life support in space. The fixtures were made from polycarbonate and CPVC plastic sheets, aluminum and stainless steel. Like the previous design, the large banks are comprised of three separate units: a power supply/control unit, a timer, and a light fixture.

### Control unit, power requirements and capability

The physical layout of the 36-Norlux HEX array control unit is shown in Figure 3C. Each LED component was independently controlled by using a pulse wave modulator (PMW; number 7 in Figure 3C) made by NorLux to control the electronic circuits for each RGB component to the desired irradiance. The controller consisted of a NEMA electrical box housing the power supply, the circuit boards and the PWM with control knobs for adjusting each circuit of HEX LED units. Each set of 30 R, G, or B dies in each HEX array was powered by a single circuit consisting of a TIP 107 PNP transistor, a 22 ohm, 1 watt resistor and a 20 ohm, 1 watt resistor assembled as in Figure 4A. This unit is repeated multiple times, one for each color on each array (Figure 4B). For each color, all of these circuits are placed in parallel and controlled by a single potentiometer. An electronic timer was used to set the photoperiod for the lights with the capability of 4 on/off programs for the complete system. This timer was purchased locally that could satisfy the voltage and ampere load requirement. The electrical power requirements were 110 volts AC and 18.72 amps maximum for all the HEX LEDs. When the HEX LEDs are operating at 100% power they are using approximately 432 watts. The entire system uses approximately 1980 watts at full operating power. At 25°C each RGB HEX LED requires 520 mA total, red 120 mA, green 200 mA, and blue 200 mA.

### The RGB 36-array bank

The aluminum, CPVC, and polycarbonate sheets were measured, cut, and assembled to create a light fixture to mount the Norlux HEX LEDs and designed to fit into custom racks within the Advance Life Sciences growth chambers at Kennedy Space Center. The custom light fixture with attached aluminum plate has dimensions of 20" × 20" × 3" (50.8 cm × 50.8 cm × 7.62 cm). The HEX LEDs were mounted directly to 0.125" (3.175 mm) aluminum sheet 20" × 20" (50.8 cm × 50.8 cm) with thermal paste to allow sufficient heat dissipation. The HEX LEDs were evenly distributed on the aluminum sheet (Figures 3D and 3E). Although the aluminum sheet was the primary heat sink for cooling the HEX arrays, secondary aluminum heat sinks were added in a vertical arrangement inside the light fixture for added cooling (Figure 3F). Three cables with 37 conductors (22 AWG) were connected from the light fixture to the circuit boards, one cable for controlling the red components (i.e., 1080 red dies) from 36 HEX LEDs, one for the green components (i.e., 1080 green dies) from 36 HEX LEDs, and one for the blue components (i.e., 1080 blue dies) from 36 HEX LEDs. The light output is approximately 150 μmoles m^-2 ^s^-1 ^at the center and approximately 85 μmoles m^-2 ^s^-1 ^at the edges when measured with a radiometer 30.5 cm vertically below from the light fixture. Fluence rates decreased at the edges, suggesting great care to be exercised in positioning of LED arrays relative to the specific application.

### Validation of LED HEX arrays using early photomorphogenic responses

The end-point hypocotyl elongation assays were performed by arranging seed in a line on a square Petri dish placed vertically under the light source. Individual seeds were placed on media containing 1 mM KCl and 1 mM CaCl_2_, they were stratified at 4°C for 48 h and then were placed into the respective conditions for 72 h. Seedlings were imaged at high resolution and hypocotyl length was measured from digital images using Image Tool (Windows Version 3.0) software. Hypocotyl kinetics in response to green light and phototropic curvature induced by blue light were performed as previously described [17,30].

## Authors' contributions

KF designed and fabricated facets of the RGB HEX array electronics and structural elements at the University of Florida (UF), performed all physiological assays and drafted the manuscript. LK designed and fabricated the HEX array platform at Kennedy Space Center (KSC). RM and JDK designed and assembled the circuit board in the UF control unit and engaged in troubleshooting the arrays. H-HK, RW and JS provided conceptual designs and oversaw the practical implementation of the technology at KSC.

## Figures and Tables

**Table 1:** The complete parts list for both array designs developed.

**Design 1 – 4 × 5 RGB Arrays**	**Design 2 – 1 × 36 RGB Arrays**
20 – NorLux HEX LED 90 Die, RGB Array, Part Number N9100-0005 4 – 14" × 18" × 3/4" aluminum blocks 4 – alloy heat sinks, approximately 5" × 3" base 2 – 12 VDC, 25 A Regulated Switching Power supply 6 – 3" (80 mm) 12 VDC fan 12 – 1 K ohm potentiometer 12 – LM317 voltage regulator with matching heat sink 12 – SPST switch 4 – 5-input RJ11 coupler on single telephone cable 12 – RJ11 connector 1 – large plastic project box 2 – terminal bus, 1 ea per power supply 2 – 110 VAC standard 24 h timer 12 – 1.0 μF capacitor 12 – 0.1 μF capacitor 12 – 1 Ω power resistor 12 – 1 K Ω resistor 1 – blank circuit board	36 – NorLux HEX LED 90 Die RGB Array, Part Number N9100-0005 1 – 20" × 20" × 1/8" sheet of aluminum 1 – 24" × 24" × 3/8" sheet of gray CPVC plastic 1 – 48" × 48" × 1/4" sheet of clear polycarbonate plastic 1 – 8" × 16" × 6" NEMA waterproof Fiberglas composite electrical box 1 – 15 VDC, 22 A Regulated Switching Power Supply 1 – 25 A 110 VAC breaker 2 – 3" (80 mm) 110 VAC fan 1 – 5" (120 mm) 15 VDC fan 36 – 22 Ω, 2 W composite resistor 72 – 20 Ω, 1 W composite resistor 108 – TIP107 NPN Regulated Transistor 108 – 4.7 K Ω 1 W composite resistor 1 – Pulse Wave Modulator (manufactured by NorLux) 3 – Blank prototype circuit board Misc. – Connectors, wire and cables, stainless steel nuts, bolts, and screws.
Telephone cable, 2-pair, 22 AWG Misc. – wire and cable, stainless steel nuts, bolts and screws.	
